# Sodium Oxybate: A Cause of Extreme Involuntary Weight Loss in a Young Lady

**DOI:** 10.1155/2019/6537815

**Published:** 2019-07-24

**Authors:** Michael G. Noujaim, Ahmad Mourad, Jeffrey D. Clough

**Affiliations:** Department of Medicine, Duke University Medical Center, 2301 Erwin Rd, 27703 Durham, NC, USA

## Abstract

We present a case of a young lady with extreme involuntary weight loss and alarming constitutional symptoms found ultimately to be all due to a single medication's side effects. The objective of this case report is to alert physicians, especially in a primary care setting, that the side effects of a medication used mostly in a highly specialized field of neurology, sodium oxybate (SXB), can cause extreme involuntary weight loss in addition to chronic night sweats and symptoms of clinical depression.

## 1. Background

Sodium oxybate (SXB) is a central nervous system depressant that primarily acts as a GABA_B_ receptor agonist [1]. SXB was approved in 2002 for the treatment of excessive daytime sleepiness and cataplexy in patients with narcolepsy in the United States [1]. Its reported adverse effects include some weight loss (<12%) and depression (3–6%) [1].

## 2. Case Report

A 27-year-old female came to our primary care clinic with 64-pound unintentional weight loss over a 10-month period. Her associated symptoms included drenching night sweats, depressed mood, a lack of interest in performing her daily activities, and decreased energy. Her medical history was significant for polycystic ovarian syndrome, dysmenorrhea, and type 1 narcolepsy with hypnagogic/hypnopompic hallucinations and cataplexy. She had been followed by neurology long term for narcolepsy and was initially treated with methylphenidate (120 milligrams daily), subsequently transitioned to SXB (9 grams daily)—given its higher efficacy—approximately 10 months prior to her presentation. During this period, she endorsed eating her baseline three meals a day. She was not exercising at all and was losing weight on a daily basis. She weighed 205 pounds at the onset of her symptoms and weighed 142 pounds on current presentation. The patient denied any gastrointestinal symptoms including nausea, vomiting, or diarrhea. Her in-clinic PHQ-9 score was 9 points. The rest of her physical exam was notable for no palpable lymphadenopathy, a normal head, heart, chest, skin, and abdominal exam. She had had a recent normal pelvic exam and a normal pap smear from the prior year. Given our patient's unexplained weight loss and constitutional symptoms, an extensive workup was performed that included computed tomography scans of her chest, abdomen, and pelvis, complete blood count, comprehensive metabolic panel, thyroid profile, anti-nuclear antibody screen, C-reactive protein, sedimentation rate, lactate dehydrogenase, urinalysis, and peripheral blood smear. All of her laboratory testing and imaging studies were normal. Given negative testing and the temporal association of her symptoms with starting SXB, we decided to hold her SXB and scheduled follow-up at 3-week intervals. On initial follow-up, the patient's night sweats and depressive symptoms had resolved, and for the first time in almost a year, she had not lost any weight but instead had gained 3 pounds.

## 3. Discussion

Weight loss, to a certain extent, is a known adverse effect of SXB. A 2008 retrospective multicenter study included 54 patients who had been using SXB for at least 3 months and found an average weight loss of 7.5 pounds, with patients with cataplexy having the most significant loss in weight [2]. Only 1 of the 54 patients in this study exhibited extreme weight loss of 68 pounds [2]. A more recent retrospective follow-up study published in 2018 found that SXB use in narcolepsy caused a significant reduction in body mass index (BMI) [3]. They reported a mean BMI decrease of 2.56 kg/m^2^ in women and 0.84 kg/m^2^ in men who had been on SXB for at least 3 months [3].

Medication adverse reactions were considered early on in our differential diagnosis. However, to our knowledge, the combination of extreme weight loss and severe constitutional symptoms due to SXB that our patient presented with has not been reported in the literature. As a result, given our patient's young age and concerning symptoms, we thought it was prudent to pursue an initial, limited yet thorough biochemical and radiological workup before trialing the patient off of SXB. Once our initial workup turned up negative, we made the decision to have our patient stop taking SXB with very close follow-up at no more than 1- to 2-week intervals. Had the patient continued to be symptomatic, we would have proceeded to more extensive testing such as positron emission tomography scanning as well as liver and bone marrow biopsies.

Using guidelines for reporting adverse drug reactions, we classify this as “dose related and time related [4].” Adverse drug reactions in this category are defined as an uncommon exaggerated pharmacologic response that are likely related to a cumulative dose of the drug with low potential for mortality [4]. Moreover, using the Naranjo algorithm for estimating the probability that this adverse reaction is due to SXB, we calculated a score of 9 points, indicating a definite link to SXB [5]. This case demonstrates the potential for SXB therapy in type 1 narcolepsy patients to cause extreme weight loss associated with other constitutional symptoms and depression. Furthermore, we highlight the importance of recognizing when to stop invasive testing and considering exaggerated adverse drug reactions in the workup of alarming symptoms in a young patient.
